# Medicine, Pathology and Therapeutics

**Published:** 1897-01

**Authors:** 


### MEDICINE, PATHOLOGY AND THERAPEUTICS.

Conducted by a member of the editorial staff.

### SENILE "PNEUMONIA."

It is a common custom among physicians (*Denver Med. Times*) to call nearly every case of acute pulmonary disorder, pneumonia, particularly if the attack is accompanied by rapid and difficult breathing and pain in the chest; yet these are cardinal symptoms of phthisical exacerbations, of pleurisy with effusion, and of pulmonary edema. Dr. L. D. White voices the well-established truth when he states that the acute lung disorders in the aged generally termed pneumonia are in reality nearly always instances of pulmonary congestion from weakened circulation, which may be dependent on feeble heart action or valvular trouble, on obstruction to the systemic circulation from renal or hepatic disease, or on general atheromatous conditions; the most prominent symptom in these cases is dyspnea. Venous obstruction is shown by livid lips and pale purple cheeks; the skin is doughy and puffy; the temperature may range high or be even subnormal; normal pulse-respiration ratio is impaired; the eye loses expression and may show *arcus senilis*. It is important in such cases to see that the bladder is emptied, and to examine the urine for renal derangement. Of all drugs, strychnine is the most efficient here. If the kidneys do not functionate, infusion of digitalis or the hot-air pack should be employed. Caffeine can be relied upon in cardiac complications. Small doses of whisky or brandy often repeated are of more value than larger amounts at infrequent intervals. The clean light pneumonia jacket of cotton wadding is far preferable to the old-time poultices. The room ought to be well ventilated and be kept at a temperature not above 60° F. Nourishment should be liquid. The bowels may be moved daily by an enema of oil and suds.

---

### TYPHOID FEVER FROM ICE CREAM.

An outbreak of typhoid fever (*Boston Med. and Surg. Journal*) occurred in the latter part of July in the town of East Barrington, N. H. The cases were all traced to a single source. The first case was an unrecognised one, the patient being unwell but helping about the house and doing part of the milking. It is supposed that he must have in some way contaminated the milk, as by going to stool and not washing his hands before returning to the milking. The water supply was carefully examined and found to be all right. On Friday evening a party was given at the house and guests ate of ice cream made at home from the milk supply referred to above. Within the next ten or fourteen days, fourteen of the guests came down with the typhoid fever—eight in the town of Barrington, of whom one died; two in Lee; one each in Dover, Rochester and Woodbury, N. H., and one in Haverhill, Mass. All of these out-of-town cases were guests at the party. No other cases occurred in the town and all were partakers of the cream.

---

### TREATMENT OF PERTUSIS.

Josias, in his new work, Therapeutique Infantile (*Universal Med. Magazine*), recommends the use of thymol instead of carbolic acid as a spray in this disease. A vessel containing the following solution is placed over a small night-lamp in the room and allowed to evaporate: ℞ Thymol, 10 grammes (2½ drachms); alcohol, 300 grammes (9½ ounces); water, 700 grammes (22½ ounces). At the Trousseau Hospital an alcoholic solution of thymol and menthol is used several times a day for spraying the rooms set apart for whooping-cough cases. A vapor-atomiser and the following formula are employed: ℞ Thymol, 6 grammes (1½ drachms); menthol, 6 grammes (1½ drachms); alcohol at 90°, 120 grammes (4

ounces). A tablespoonful in the atomiser, which has been previously filled with water.

---

CONTINUED FEVER, NOT TYPHOID.

RECENTLY J. M. Da Costa related (*Phila. Polyclinic*) several cases of continued fever of long duration, differing thus from the ordinary type, which he had no hesitation in diagnosticating as such to the exclusion of typhoid fever. Every physician of large experience meets with such cases. In his earlier practice, doubting perhaps his own ability, he manages them on a "probable diagnosis" of typhoid, and does, at least, no harm. In recent years such cases have been, doubtless, termed "influenza," just as in earlier years genuine cases of catarrhal fever were termed typhoid; yet they possess still less in common with influenza than with enteric fever. At present in Philadelphia such cases are more common than at any former time. They are not influenza, not abortive typhoid, and while it is exceptional now as formerly to find cases of the long duration described by Da Costa, yet the tendency to long continuance in the absence of treatment is marked.

Cases differ in semeiology almost as much as patients differ in physiognomy; they are doubtless of varying etiology and mechanism, yet an attempt at strict classification would be as futile as such refinement in diagnosis is unnecessary.

What is needed is to exclude the recognised specific infections (including malaria), and, this being done, to manage the case on general principles, as one of acute intoxication. The toxic agent may have been introduced from without, in food or water, or may have been generated within the body as a result of the chemic or biologic action of an agent from without, or through failure of physiologic function. The cause and process cannot always be traced. The treatment is to put the patient at rest, cleanse the *primæ viæ*, regulate the diet, promote comfort by sponging with cool or tepid aromatised water, and give drugs only in the presence of some definite indication. Usually such a definite indication is given by the chaiacter of the stools, and an insoluble "intestinal antiseptic" is of service. Sometimes a general tonic, such as strychnine or quinine in small dcses, is called for. Alcohol is rarely needed. Coincident bronchitis may require the exhibition of a terebinthinate or an ammonium salt. However, as before noted, drugging is not urgent, and must be a matter of good judgment.

